# Magnetic resonance spectroscopy and auditory brain-stem response audiometry as predictors of bilirubin-induced neurologic dysfunction in full-term jaundiced neonates

**DOI:** 10.1007/s00431-023-05246-z

**Published:** 2023-11-18

**Authors:** Lamiaa Khaled Zidan, Mohamed Ahmed Rowisha, Mohammed Abd Ellatif Nassar, Rasha Ahmed Elshafey, Trandil Hassan El Mahallawi, Heba Saied Elmahdy

**Affiliations:** 1 Pediatric Department, Faculty of Medicine, Tanta University, Tanta, Egypt; 2 Radiology Department, Faculty of Medicine, Tanta University, Tanta, Egypt; 3 Audiology Department, Faculty of Medicine, Tanta University, Tanta, Egypt

**Keywords:** Neonatal hyperbilirubinemia, Bilirubin encephalopathy, MRS, ABR, BIND

## Abstract

The purpose of this research was to define the functions of MRS and ABR as predictors of bilirubin-induced neurologic dysfunction (BIND) in full-term neonates who required intervention (phototherapy and/or exchange transfusion**).** This prospective cohort study was done at the NICU of Tanta University Hospitals over a 2-year duration. Fifty-six full-term neonates with pathological unconjugated hyperbilirubinemia were divided according to MRS and ABR findings into 2 groups: group (1) included 26 cases with mild acute bilirubin encephalopathy (BIND-M score 1–4). Group (2) included 30 cases with neonatal hyperbilirubinemia only. In addition, 20 healthy neonates with similar ages were employed as the controls. When compared to group 2 and the control group, group 1’s peak-area ratios of NAA/Cr and NAA/Cho were found to be significantly reduced (*P* < 0.05). As compared to group 2 and the control group, group 1’s Lac/Cr ratio was significantly greater (*P* < 0.05), but the differences were not significant for group 2 when compared to the control group. Waves III and V peak latencies, I–III, and I–V interpeak intervals were significantly prolonged in group 1 in comparison to group 2 and controls (*P* < 0.05) with no significant difference between group 2 and control group.

*Conclusion*: When the symptoms of ABE are mild and MRI does not show any evident abnormalities, MRS and ABR are helpful in differentiating individuals with ABE from patients with neonatal hyperbilirubinemia.

*Trial registration*: ClinicalTrials.gov, Identifier: NCT06018012.

**Table:** 

**What is Known:** *• MRS can be used as a diagnostic and prognostic tool for the differential diagnosis of patients with acute bilirubin encephalopathy, from patients with neonatal hyperbilirubinemia*	
**What is New:** *• ABR is a useful diagnostic and prognostic tool in the care and management of neonates with significantly raised bilirubin. It can be used as early predictor of acute bilirubin encephalopathy in the earliest stage of auditory damage caused by bilirubin.*

**What is Known:**

*• MRS can be used as a diagnostic and prognostic tool for the differential diagnosis of patients with acute bilirubin encephalopathy, from patients with neonatal hyperbilirubinemia*

**What is New:**

*• ABR is a useful diagnostic and prognostic tool in the care and management of neonates with significantly raised bilirubin. It can be used as early predictor of acute bilirubin encephalopathy in the earliest stage of auditory damage caused by bilirubin.*

## Introduction

More than 50% of all newborns have neonatal jaundice during the first 3 to 5 days after birth, making it one of the most prevalent conditions. It is normally a benign transitional phenomenon after birth, expressing a dynamic balance between the synthesis and elimination of bilirubin. The risk of developing possibly harmful hyperbilirubinemia and bilirubin encephalopathy in infants is significantly elevated due to increased bilirubin production and delayed bilirubin clearance [1].

The syndrome of bilirubin-induced neurological dysfunction (BIND) is a spectrum of neurological symptoms in newborns who have been exposed to high amounts of bilirubin. So early detection of this state can eliminate long-standing and irreversible brain damage [2].

Although the BIND spectrum embraced kernicterus, acute bilirubin encephalopathy, and isolated neural pathway dysfunction [3], BIND is a term whose meaning has evolved over time.

In its current usage, BIND characterizes a constellation of more subtle neurodevelopmental disabilities without the classical clinical findings of kernicterus. It includes disturbances of central auditory processing, coordination, muscle tone, and sensorimotor integration [4].

Some of the neurotoxic effects of bilirubin are gliosis, demyelination, and inhibition of glutamate (Glu) uptake by astrocytes in the basal ganglia [5]. Magnetic resonance spectroscopy (MRS) is a specialized imaging procedure that should be able to detect these metabolic changes, having considerable promise for both early detection and long-term monitoring of hyperbilirubinemia in neonates [6].

The auditory system is sensitive to the neurotoxicity of bilirubin. In fact, it is believed that the cochlear nuclei of the brainstem are one of the first structures impacted by a high total bilirubin level, followed by the auditory nerve [7]. This harm may happen in a lack of other classic bilirubin encephalopathy signs and is recognized as auditory neuropathy spectrum disorder (ANSD), which is often characterized by impaired auditory neural function (altered or absent auditory brainstem response audiometry (ABR) waveforms) in the existence of normal cochlear microphonics and/or cochlear otoacoustic emission [8].

These auditory implications might vary from slight speech and hearing processing impairments to total deafness, which is perceived by ABR audiometry; hence, an abnormal ABR result signifies the presence of acute bilirubin encephalopathy (ABE) and is considered the most common and the earliest manifestation of ABE [7].

In this study, we hypothesized that neonates with high bilirubin levels, even not yet in the exchange transfusion zone, might have neurological compromise, which can be detected early by MRS and ABR.

## Patients and methods

This prospective cohort research was authorized by the ethics council of the Faculty of Medicine at Tanta University, Egypt, with approval number (32886/01/19). The informed permission of the parents of all babies recruited was obtained. Enrollment criteria included full-term neonates (gestational age 37 to 42 weeks) who developed pathological unconjugated hyperbilirubinemia that necessitated therapeutic intervention (intensive phototherapy and/or exchange transfusion) using the American Academy of Pediatrics guidelines; 2004. In order to compare the neurological imaging and hearing test results, a control group consisting of 20 full-term, appropriate for gestational age (AGA), and healthy neonates was recruited.

### Study population and setting

**Fig. 1:**
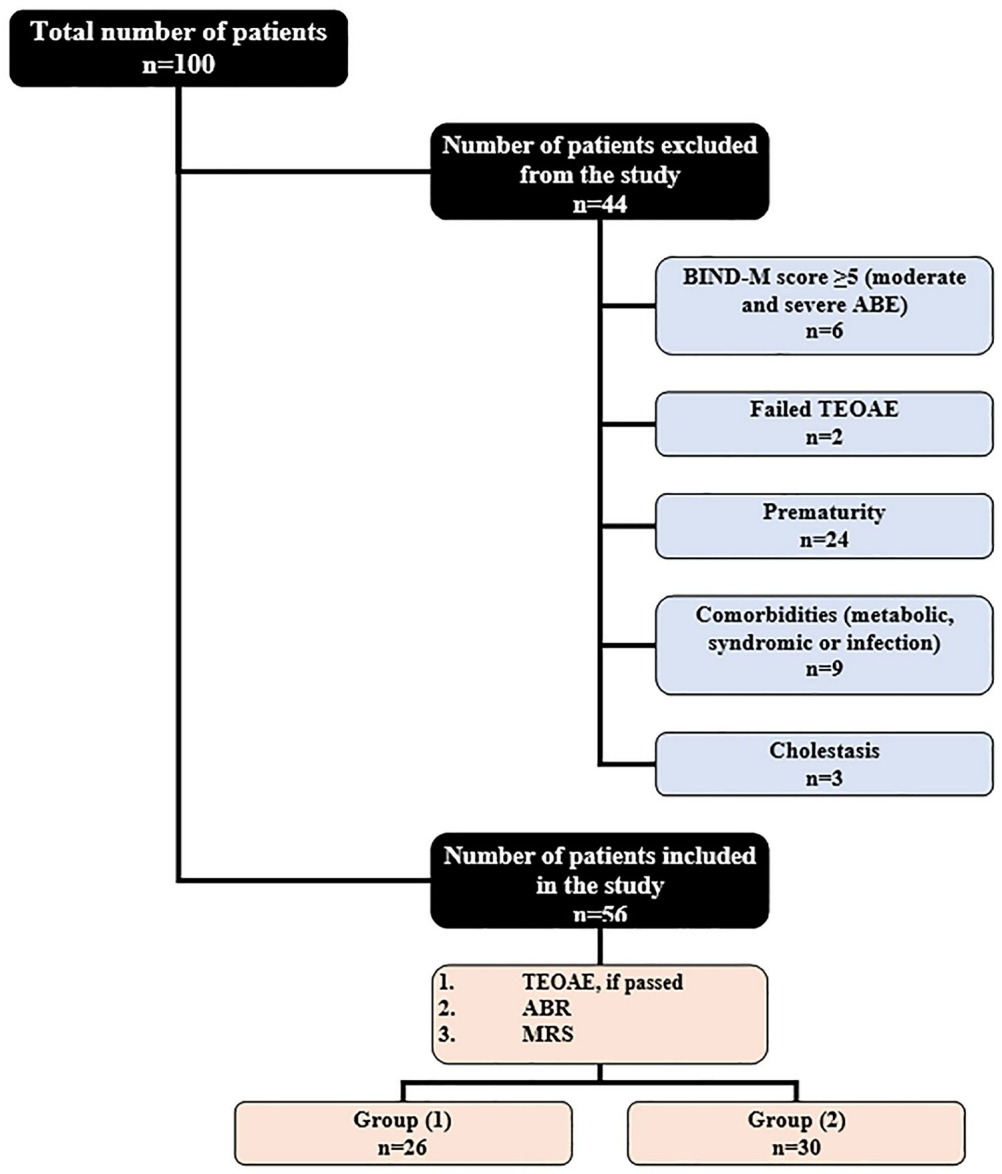
Study population

Patients were enrolled from March 2019 to March 2021. During the research period, 100 newborns were brought into the neonatal intensive care unit (NICU) of Tanta University Children’s Hospital, which requires intervention for unconjugated hyperbilirubinemia (whether exchange transfusions and/or phototherapy), of whom 56 term infants were enrolled in the study and the other 44 infants were excluded (Fig. 1) according to exclusion criteria that included preterm neonates (less than 37 weeks), clinically moderate and severe ABE according to modified bilirubin-induced neurologic dysfunction (BIND-M) score (5–9) [9]; neonates who were born with birth asphyxia and/or had a low Apgar score; neonates with sepsis including central nervous system (CNS) infection; neonates with family history of childhood hearing loss, congenital infection, chromosomal abnormalities, congenital ear anomalies associated with hearing loss or brain, and abnormalities including craniofacial anomalies; patients who were receiving ototoxic drugs as aminoglycosides and conjugated hyperbilirubinemia.

### Neonatal evaluation

Following admission and enrollment, full antenatal, perinatal, and postnatal history were taken together with a thorough clinical examination. Gestational age and admission weight were documented. The neurological assessment involved the modified bilirubin-induced neurological dysfunction (BIND-M) score, which was performed for all babies. BIND-M score is used to detect and grade the presence of acute bilirubin encephalopathy by evaluating mental status, muscle tone, cry pattern, and oculomotor or eye movements. Scores of (1–4), (5–6), and (7–9) indicate mild, moderate, and severe acute bilirubin encephalopathy (ABE), respectively [9].

### Laboratory investigations and treatment

Total serum bilirubin (TSB) was measured at admission and repeated 4–6 h after starting the treatment, and 12–24 hourly when bilirubin became stable or falling, till discontinuation of phototherapy using the Synchrou Cx9 Pro automated Beck-man coulter (TX, USA). In addition, a complete blood count and reticulocyte count were performed for all enrolled jaundiced newborns. Both mothers and babies were tested for ABO and Rh blood groups. Coomb’s test was performed whenever available. Also, serum albumin and bilirubin/albumin ratio were done on admission. A decision for phototherapy or exchange transfusion and phototherapy was made according to the protocol of our unit, which follows guidelines of the American Academy of Pediatrics for neonatal hyperbilirubinemia therapy (2004) [10].

### ABR and transient evoked otoacoustic emissions (TEOAE) protocol

Each participant underwent an otorhinolaryngological examination before the evaluation process using the ABR and TEOAE tests to rule out any middle ear pathologies through inspection of the external acoustic canal (to avoid any obstruction of the meatus or collapsing the acoustic lumen), (using Immittacemetry of Interacoustics AT235h, Madsen, Denmark). It includes acoustic reflex and high-frequency tympanometry. Both procedures were carried out after the decline of TSB to the discharge level and just before discharge from the hospital in a quiet, dimly lit, and electrically shielded room at Tanta University Hospital’s ENT department’s Audiology unit, during quiet sleep after feeding to avoid agitation and excessive muscle activity, which may cause artifacts.

All infants with acoustic reflex and normal tympanometric pressures underwent TEOAE. It was done by monitoring changes in TEOAE and utilizing the intelligent hearing system’s otoacoustic emissions to record the results as (pass–fail) (IHS, Miami, USA). All recruited neonates had normal (TEOAE).

ABR was recorded with SmartEPs (TM, Intelligent Hearing system, Miami, USA). A positive electrode was placed up on the forehead, and a negative electrode was placed on the ipsilateral mastoid, and a ground electrode was placed on the contralateral mastoid. Stimuli for ipsilateral clicks were provided with TDH 49 headphones at 90 dBnHL, alternating polarity, and a repetition rate of 19.3 s. The stimulus intensity started at 90 dBnHL down to 30 dBnHL or to the threshold. Absolute (I, III, and V) wave latencies and interpeak latencies (I–III, I–V, III–V) were recorded and compared between case groups relative to the control group. The presence of repeatable wave V at the Intensity of 30 dBnHL was taken as the normal threshold. ABR measures considered for the diagnosis of abnormality were the absence of ABR waves, loss of one or more peaks of I–V at 90 dBnHL, raised threshold, prolonged absolute latencies of wave I, III, and V peaks, or prolonged interpeak intervals of I–III, III–V, and I–V.

### MRI and MRS protocol

The MRI and MRS examinations were performed in all the neonates after declining of TSB to the discharge level and just before discharge from the hospital in one session, after all guardians were informed about the MRI magnets effect, and the approximate duration of MRI and MRS techniques, utilizing a whole-body MR imager and a 1.5-T General Electric system (SIG-NATM Explorer, GE Healthcare Systems, Chicago, USA). Magnetic resonance imaging (MRI) was performed on all of the participants, with routine sequences including axial fast spin echo T2-weighted (3000/91/1), axial T2-weighted (300/14/1 (TR/TE/excitations)), axial T1-weighted (500/14/1), axial fast fluid attenuation inversion recovery (FLAIR) (10002/172/1, T1 2.2 s), and diffusion-weighted images (DWI) obtained with an axial single shot echoplanar spin-echo sequence (TR/TE: 10000/89.9– 99.3).

In MRS, Axial FLAIR imaging was used for voxel localization. The basal ganglia, which were carefully examined to prevent interference from the skull bone and CSF fluid, were the focus of interest in all infants. The voxel size ranged from 1.5 × 1.5 × 1.5 to 2 × 2 × 2 cm^3^. The average acquisition time was 5 min and 4 s for each spectral acquisition and 20 min overall. Chemical shift imaging, sometimes referred to as spectroscopic imaging (MRSI), was utilized as a localization method. Point-resolved spectroscopy (PRESS) was the kind of pulse sequence that was employed, and the parameters were long TE (TR/TE = 2000/144 ms) and short TE (TR/TE = 2000/35 ms). The data were then post-processed on a GE Advantage workstation, where the magnitude spectra were automatically corrected for baseline and fitted with curves in order to establish the resonance regions of individual metabolites. Long TE was utilized to clearly show the intensity peak of Cho, NAA, and Cr, as well as to determine the ratio of NAA to Cr, NAA to Cho, and Cho to Cr. The primary purpose of short TE was to demonstrate the Lac and Glx peaks.

According to ABR and MRS findings, the enrolled 56 jaundiced neonates were divided into 2 groups: group (1) included 26 cases with bilirubin-induced neurologic dysfunction (acute bilirubin encephalopathy (ABE) group). Group (2) included 30 cases with only neonatal hyperbilirubinemia (NH group).

### Statistical data

The IBM SPSS software program version 20.0 was used to do the analysis once the data were entered into the computer (Armonk, NY: IBM Corp). The qualitative data were represented numerically (percentage), and comparisons were made using either the chi-square (*X*^2^) test or Fisher’s exact test, depending on the circumstance. The mean, standard deviation (SD), range (minimum and maximum), median, and interquartile range (IQR) were the statistical measures that were used to characterize the quantitative data. At the 5% level, the significance of the findings that were obtained was assessed. *t*-test and Mann–Whitney test were used to compare the results of the two groups that were analyzed. When comparing more than two groups, the *F*-test (also known as ANOVA) was used for those with normally distributed quantitative parameters. The post hoc test (also known as Tukey) was used when comparing only two groups. The post hoc test (Dunn’s multiple comparisons test) was used for pairwise comparisons, while the Kruskal–Wallis test was utilized for abnormally distributed quantitative parameters in order to compare between more than two investigated groups. The diagnostic performance of the test is represented by the amount of space that is under the ROC curve. Performance that is over 50% is considered acceptable, while performance that is close to 100% is considered the greatest possible performance for the test.

## Results

BIND-M score on admission (median = 2) was significantly higher in group 1. Although being statistically insignificant (*P* value = 0.356), gender among the studied groups showed male predominance as 61.5% of group 1 were males and females made up 38.5% (the ratio of male to female was at 1.6:1). Similarly, group 2 enrolled 76.7% male neonates and 23.3% female neonates. Jaundice presented significantly earlier in neonates of group 1 than those of group 2. In comparison to group 2, group 1’s hospital stay (days) lasted longer.

Maximum TSB on admission (mg/dl) was significantly higher in group 1. Hemoglobin was significantly lower in group 1 than in group 2, and reticulocyte count (%) was significantly higher in group 1 than in group 2. The bilirubin/albumin ratio (B/A ratio) on admission was significantly higher in group 1 than in group 2 (Table 1).

**Table 1:** Demographic and clinical data of the studied groups

	**ABE group (*n* = 26)**		**NH group (*n* = 30)**		**Control group (*n* = 20)**		**Test of Sig.**	***P***
	**No.**	**%**	**No.**	**%**	**No.**	**%**		
**Gender**								
Male	16	61.5	23	76.7	12	60.0	*χ*^2^ = 2.065	0.356
Female	10	38.5	7	23.3	8	40.0		
**Gestational age (weeks)**								
Min.–max.	37.0–39.0		37.0–40.0		37.0–40.0		*F* = 0.397	0.673
Mean ± SD	37.96 ± 0.66		38.17 ± 0.83		38.10 ± 1.12			
**Weight (kg)**								
Min.–max.	2.60–3.50		2.80–3.80		2.90–3.60		*F* = 0.837	0.437
Mean ± SD	3.07 ± 0.27		3.11 ± 0.51		3.21 ± 0.19			
**Age on admission (hours)**								
Min.–max.	12.0–108.0		36.0–120.0				*t* = 2.947^*^	< 0.001^*^
Mean ± SD	52.57 ± 25.52		72.0 ± 23.79					
**Duration of hospitalization (days)**								
Min.–max.	6.0–15.0		5.0–11.0				*t* = 6.282^*^	< 0.001^*^
Mean ± SD	10.69 ± 2.90		6.75 ± 1.46					
**BIND-M score on admission**								
Median (IQR)	2 (1–3)		1 (1–1)				*U* = 160.50^*^	< 0.001^*^
Min.–max.	1.0–3.0		0.0–2.0					
**Maximum TB on admission (mg/dl)**								
Min.–max.	16.0–26.0		17.0–22.50				*t* = 3.286^*^	0.002^*^
Mean ± SD	20.65 ± 3.0		18.54 ± 1.39					
**B/A ratio on admission**								
Min.–max.	4.30–7.10		4.10–6.10				*t* = 4.151*	< 0.001^*^
Mean ± SD	5.81 ± 0.82		5.04 ± 0.52					

*SD* standard deviation, *P P* value for comparing between the studied groups, *χ*^2^ chi-square test, *F F* for ANOVA test, *U* Mann–Whitney test, *B/A* ratio bilirubin/albumin ratio

^*^Statistically significant at *P* ≤ 0.05

Hyperintensity of globus pallidus is considered a radiological sign of bilirubin encephalopathy; however, on T1-weighted MRI, only 8 (30.8%) cases in group 1 displayed unusually elevated signal intensity across the globus pallidus (Table 2).

**Table 2:** MRI findings of the studied groups (at days 6–10)

**Hyperintensity of globus pallidus (T1 W1)**	**ABE group (*n*** **= 26)**		**NH group (*n*** **= 30)**		***χ***2	^**FE**^***P***
	**No.**	**%**	**No.**	**%**		
Absent	18	69.2	30	100.0	10.769	0.001^*^
Present	8	30.8	0	0.0		

*SD* standard deviation, *P P* value for comparing between the studied groups, *χ*^2^ chi-square test, *FE* Fisher’s exact test

^*^Statistically significant at *P* ≤ 0.05

In comparison to groups 2 and the control group, group 1’s NAA/Cr and NAA/Cho peak ratios were significantly lower, whereas group 2’s lowered ratios were statistically insignificant when compared to the control group. However, the Lac/Cr ratio was statistically insignificantly elevated in group 2 when compared to the control group, whereas it was significantly higher in group 1 when compared to groups 2 and control group (Table 3).

**Table 3:** MRS metabolic ratios in the basal ganglia of the studied groups

	**ABE group (*n* = 26)**	**NH group (*n* = 30)**	**Control group (*n* = 20)**	**Test of Sig.**	***P***
**NAA/Cr**					
Min.–max.	0.50–1.03	0.95–1.35	1.01–1.40		< 0.001^*^
Median (IQR)	0.72 (0.65–0.83)	1.12 (1.0–1.3)	1.20 (1.1–1.3)	*H* = 50.546^*^	
**Sig. bet. groups**	*P*_1_ < 0.001^*^, *P*_2_ < 0.001^*^, *P*_3_ = 0.731				
**NAA/Cho**					
Min.–max.	0.50–0.70	0.50–1.0	0.70–1.10	*F* = 33.668^*^	< 0.001^*^
Mean ± SD	0.62 ± 0.06	0.79 ± 0.09	0.81 ± 0.11		
**Sig. bet. groups**	*P*_1_ < 0.001^*^, *P*_2_ < 0.001^*^, *P*_3_ = 0.832				
**Lac/Cr**					
Min.–max.	0.70–1.70	0.10–0.50	0.01–0.25		
Median (IQR)	1.10 (1.05–1.5)	0.2 (0.10–0.35)	0.1 (0.05–0.19)	*H* = 53.660^*^	< 0.001^*^
**Sig. bet. groups**	*P*_1_ < 0.001^*^, *P*_2_ < 0.001^*^, *P*_3_ = 0.127				

*IQR* interquartile range, *P*_1_ *P* value for comparing between group 1 and group 2, *P*_2_ *P* value for comparing between group 1 and control, *P*_3_ *P* value for comparing between group 2 and control, *H H* for Kruskal–Wallis test; pairwise comparison bet. every 2 groups were done using post hoc test (Dunn’s for multiple comparisons test), *F* *F* for one-way ANOVA test; pairwise comparison bet. every 2 groups were done using post hoc test (Tukey)

^*^Statistically significant at *P* ≤ 0.05

In comparison to group 2 and the control group, group 1’s wave V peak latency, ABR wave III peak latency, I-V interpeak interval, and I-III interpeak interval were all significantly longer than those of group 2 and the control group, with no significant variation between group 2 and control group (Table 4).

**Table 4:** ABR wave latencies (ms) and interpeak intervals (ms) in the studied groups

	**ABE group (*n* = 21)**	**NH group (*n* = 30)**	**Control group (*n* = 20)**	**Test of Sig.**	***P***
**Wave III (ms)**					
Min.–max.	4.60–7.78	4.48–4.66	4.40–4.72		< 0.001^*^
Median (IQR)	4.72 (4.7–4.8)	4.61(4.6–4.6)	4.59 (4.5–4.6)	*H* = 29.850^*^	
**Sig. bet. groups**	*P*_1_ < 0.001, *P*_2_ < 0.001, *P*_3_ = 0.410				
**Wave V (ms)**					
Min.–max.	7.30–7.80	6.81–7.30	6.69–7.31	*F* = 48.590^*^	< 0.001^*^
Mean ± SD	7.51 ± 0.12	7.15 ± 0.13	7.11 ± 0.19		
**Sig. bet. Groups**	*P*_1_ < 0.001, *P*_2_ < 0.001, *P*_3_ = 0.634				
**I-III interpeak (ms)**					
Min.–max.	2.69–3.59	2.63–3.29	2.59–3.21	*F* = 21.619^*^	< 0.001^*^
Mean ± SD	3.26 ± 0.32	2.85 ± 0.20	2.84 ± 0.20		
**Sig. bet. groups**	*P*_1_ < 0.001, *P*_2_ < 0.001, *P*_3_ = 0.980				
**I-V interpeak (ms)**					
Min.–max.	5.30–5.55	4.80–5.46	4.50–5.80	*F* = 14.835^*^	< 0.001^*^
Mean ± SD	5.46 ± 0.06	5.26 ± 0.14	5.24 ± 0.21		
**Sig. bet. groups**	*P*_1_ < 0.001, *P*_2_ < 0.001, *P*_3_ = 0.860				

*IQR* interquartile range, *P*_1_ *P* value for comparing between group 1 and group 2, *P*_2_ *P* value for comparing between group 1 and control, *P*_3_ *P* value for comparing between group 2 and control, *F F* for one-way ANOVA test; pairwise comparison bet. every 2 groups were done using post hoc test (Tukey), *H H* for Kruskal–Wallis test; pairwise comparison bet. every 2 groups were done using post hoc test (Dunn’s for multiple comparisons test)

^*^Statistically significant at *P* ≤ 0.05

The most prevalent ABR anomaly detected in group 1 was an elevated auditory threshold (100%). The commonest affected wave was wave V (69.2%) followed by wave III (57.5%). The commonest affected interpeak interval was the I–V interval (61.5%) followed by I–III (52%). Only 5 (19.2%) cases showed absent ABR waveforms, and all of them had TSB levels above 20 mg/dl. The minimum TSB level for ABR affection was 16 mg/dl, which affected the auditory threshold (raised) and I–III interpeak interval (prolonged).

**Fig. 2:**
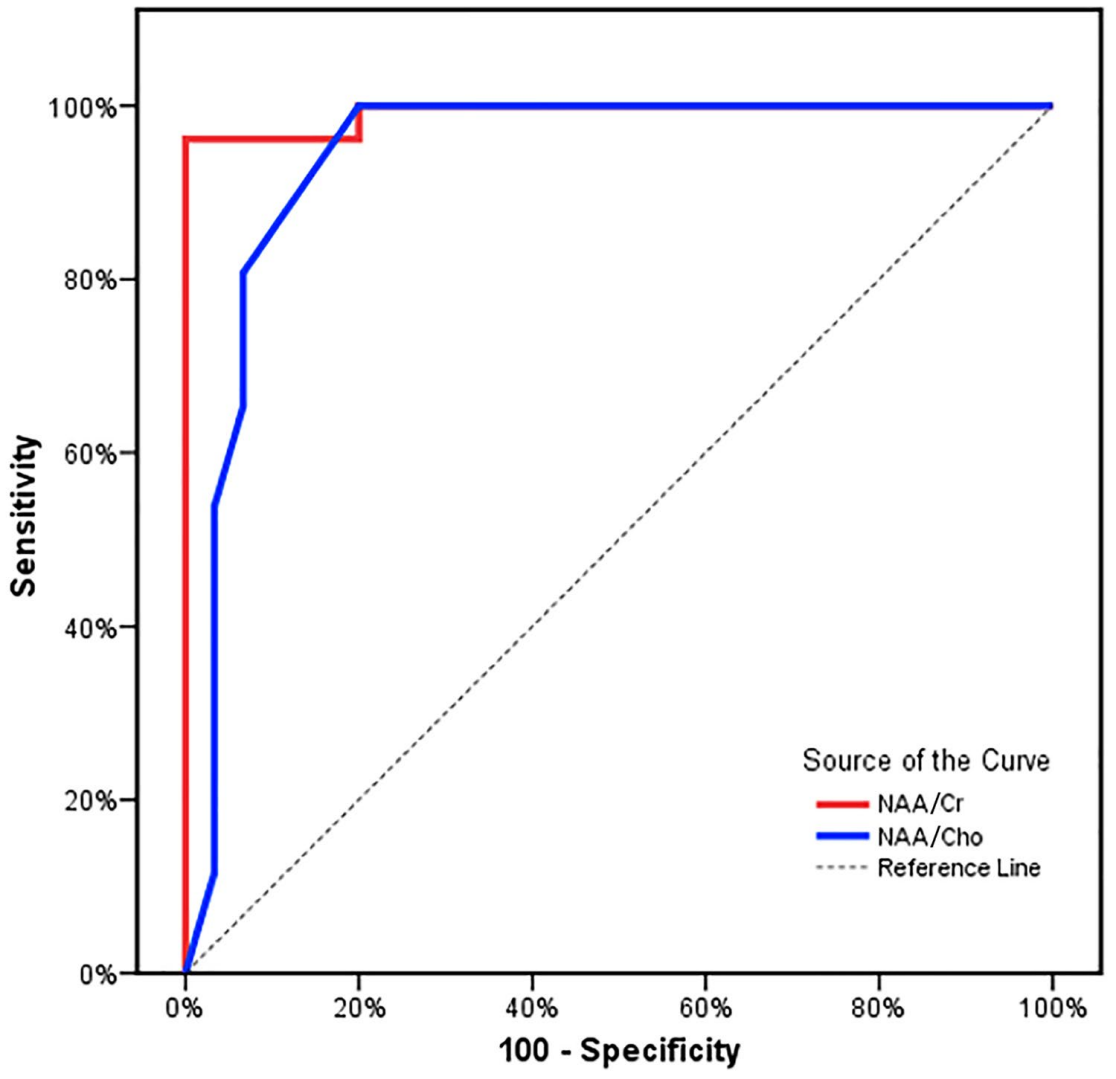
ROC curve evaluating the sensitivity and specificity of NAA/Cr and NAA/Cho for detection of ABE

Using ROC analysis, the diagnostic ability of the MRS peak ratios (NAA/Cr and NAA/Cho), to correctly distinguish between those who had mild ABE and those who did not, was found to be excellent, with an AUC of 0.992 and 0.942 for NAA/Cr and NAA/Cho, respectively. Cut-off values of ≤ 0.95 and ≤ 0.68 for NAA/Cr and NAA/Cho correspondingly yielded a sensitivity of 96.15% and 80.77%, respectively, and specificity of 96.67% and 93.33% correspondingly, and the positive predictive values (PPV) were 96.2% and 91.3% correspondingly, whereas the negative predictive values (NPV) were 96.7% and 84.4% correspondingly (Fig. 2).

**Fig. 3:**
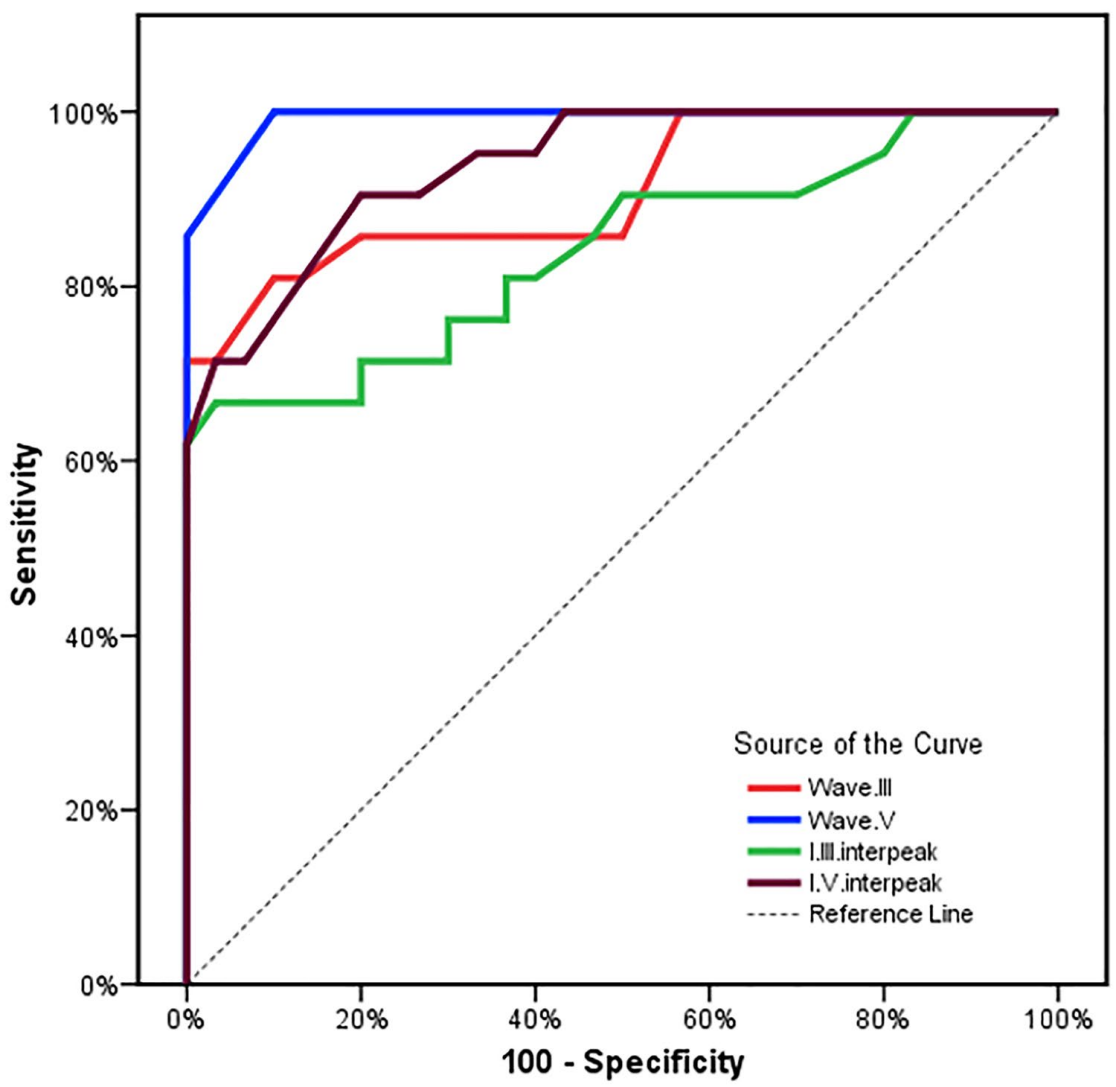
ROC curve evaluating sensitivity and specificity of ABR waves and interpeak intervals for detection of ABE

The ROC curve analyzing wave V peak latencies and interpeak I-V intervals for both specificity and sensitivity demonstrated that cut-off values of > 7.29 ms and > 5.37 ms, respectively, yielded a sensitivity of 100% and 90.48%, respectively, with specificity of 90% and 80%, respectively. PPV were 87.5% and 76%, respectively, and NPP were 100% and 92.3%. The area under the curve (AUC) for wave V peak latency and I–V interpeak interval were 0.993 and 0.939, respectively; thus, they were good at distinguishing between those who had mild ABE and those who did not (Fig. 3).

## Discussion

Symptoms of bilirubin encephalopathy can either be slight or nonexistent, and newborns with the disorder may not always present definitive neurological signs throughout the neonatal period, making bilirubin encephalopathy difficult to diagnose clinically [11–13]. Predicting bilirubin-induced neurotoxicity, particularly in sick preterm infants, based just on serum bilirubin levels is insufficient. Therefore, there is a need for a fast and non-invasive method to detect impending cell damage and neurotoxicity [14–17]

This research aimed to define the function of MRS and ABR audiometry for the distinction between infants with mild ABE and infants with hyperbilirubinemia only without ABE who were hospitalized in the Neonatal Intensive Care Unit (NICU), Pediatrics department, Tanta University Hospitals over two years duration.

This research demonstrated a substantial variance in maximal TSB at admission across the investigated groups. (mg/dl) (mean ± SD = 20.65 ± 3.0 and 18.54 ± 1.39 in group 1 and group 2, respectively), with P value = 0.002. Also, the B/A ratio on admission (Mean ± SD) was significantly higher (5.81 ± 0.82 mg/g) in group 1 than in group 2 (5.04 ± 0.52 mg/g).

Aly and Montaner [14] found that maximum bilirubin (mean ± SD) in the ABE group was 36.21 ± 7.96 mg/dl, and in the neonatal hyperbilirubinemia group was 27.78 ± 1.95 mg/dl, with significant P value (0.003).

Wu et al. [18] recruited 11 individuals with neonatal bilirubin encephalopathy, 8 individuals with neonatal hyperbilirubinemia, and 9 healthy, matched in age neonates as controls, revealed that maximum bilirubin (mean ± SD) in bilirubin encephalopathy group was insignificantly higher (31.3 ± 7.4 mg/dl) than in neonatal hyperbilirubinemia group (25.4 ± 4 mg/dl), P value was 0.075.

Our results coincide with Aly and Montaser [14] and Wu et al. [18] regarding higher TSB in the ABE group, although their values were higher than values in the current study, as both studies only recruited neonates with TSB higher than 20 mg/dl [14, 18].

Although hyperintensity of globus pallidus is considered a radiological sign of bilirubin encephalopathy, only 30.8% of cases in group 1 had an abnormally increased signal intensity on T1-weighted MRI in the globus pallidus. This agrees with Aly and Montaser [14], who reported only 16% of the ABE group showed an excessively high signal intensity in the globus pallidus. Also, our results agree with Sari et al*.* [19], who found that only 22.2% of their ABE group had abnormal MRIs. Thus, normal MRI does not exclude ABE.

On the other hand, Early magnetic resonance imaging of at-risk infants (either at or soon after ABE), while frequently showing increased T1-signal in these regions, may give false positive findings due to the presence of myelin in these structures. Advanced imaging, such as magnetic resonance spectroscopy, may shed new insights into the pathogenesis of bilirubin-induced brain injury [20].

There are several areas where the application of MRS techniques in neonates with bilirubin encephalopathy could further elucidate the pathogenesis of cellular injury. One of the most important areas pertains to energy metabolism. It has been hypothesized that the pathogenesis of cell damage in kernicterus involves bilirubin acting directly upon mitochondria, uncoupling oxidative phosphorylation, and inhibiting the ADP/inorganic phosphate reaction [3]. The uncoupling of oxidative phosphorylation, in turn, results in a dramatic decrease in ATP concentrations and a concomitant increase in anaerobic metabolism, with a marked increase in lactate production. MRS is a well-established technique for demonstrating energy failure and anaerobic metabolism in neonates with perinatal brain injury [21].

Although requiring additional hardware and software relative to conventional H^1^MRS, ^31^P-MRS could provide a direct assessment of oxidative metabolism and ATP concentration in neonates with ABE. Moreover, even without the added value of ^31^P-MRS, conventional H^1^MRS could still demonstrate much about energy metabolism in neonates with ABE. For example, the synthesis of NAA, as well as glutamate, is tightly coupled to oxidative metabolism, and thus, monitoring NAA concentrations may provide valuable information regarding mitochondrial metabolism in neonates with ABE [20].

Consequently, the ratio of NAA/Cr is regarded as a metabolic indicator of the functional condition of axons and neurons in the brain; hence, it declines in cases of neuronal or axonal degeneration or malfunction [21]. In this research, peak-area ratios of NAA/Cr and NAA/ Cho in the basal ganglia were substantially reduced in group 1 compared with group 2 and the control group, with statistically insignificant decreased ratios in group 2 in comparison with the control group. This may have been caused by the loss of neurons and gliosis in the basal ganglia of ABE patients.

These findings are in agreement with those of Wu et al. [18] NAA/Cr and NAA/Cho ratios in the basal ganglia were considerably lower in neonatal bilirubin encephalopathy patients than in neonatal hyperbilirubinemia and control patients (P < 0.05). NAA/Cr and NAA/Cho in the basal ganglia were lower in the neonatal hyperbilirubinemia group compared to the control groups, although the variations were not statistically significant. Also, our results were also consistent with Aly and Montaser [14], who found the ratio of NAA/Cr in the basal ganglia was significantly less for the ABE group compared with neonatal hyperbilirubinemia group.

Oakden et al. [22] assessed six newborns with neonatal bilirubin encephalopathy and found no changes for NAA/Cr in individuals in the basal ganglia compared to the control group of published research, which opposes our results. The reason for the difference with Oakden et al. [22] may be related to the fact that they compared their participants to the control group of study [23] that had been published, while our study included a control group that was healthy.

Regarding the NAA/Cho peak ratio in basal ganglia, our results are also consistent with Groenendaal [24], who evaluated five neonates suffering from severe hyperbilirubinemia (2 neonatal bilirubin encephalopathy individuals, 3 neonatal hyperbilirubinemia individuals) and reported lower NAA/Cho ratio in the basal ganglia of neonates with neonatal bilirubin encephalopathy indicating neuronal damage. Similarly, our study agrees with Wang et al. [25] who investigated 24 neonates with neonatal bilirubin encephalopathy and administered ^1^H-MRS to 9 of the 24 participants as well as 7 matched in age-healthy controls. They revealed that in the basal ganglia, the NAA/Cho ratio was significantly lower (P < 0.05) in the patients than in the controls.

As stated before, lactate is a byproduct of anaerobic glycolysis. Therefore, conditions involving anaerobic metabolism, which includes ischemia, cerebral hypoxia, seizures, and metabolic disorders, particularly mitochondrial ones, cause a rise in lactate concentration.; however, small amounts of lactate may be present in neonates, normally [26].

Although it is still completely unclear, there is evidence that bilirubin is harmful to mitochondria. The mitochondrial membrane was disrupted by bilirubin, which resulted in increased permeability, reduced potential, the release of cytochrome C, as well as the induction of apoptosis [27]. Evaluating Lac/Cr ratio among the full-term neonates who were enrolled in this current study with only indirect hyperbilirubinemia, without any other pathological conditions that could affect this ratio as HIE or metabolic diseases, we found that Lac/Cr ratio was substantially higher in group 1 than group 2 and control group, with statistically insignificant elevation in group 2 when compared with the control group.

Groenendaal et al. [24] studied the MRS in 5 newborns with severe hyperbilirubinemia and found that two of them had unusually elevated lactate-to-N-acetyl aspartate ratios (Lac/NAA). On MRI, one of them had basal ganglia abnormalities, and during the follow-up, the other newborn revealed neurological abnormalities. Alteration in the function of mitochondria may have contributed to the elevated Lac/NAA ratio. Consequently, the elevated Lac/Cr ratio in our study supports that bilirubin may actually cause changes in mitochondrial function that lead to a decreased aerobic metabolism.

Using ROC analysis, the diagnostic ability of the MRS peak ratios (NAA/Cr and NAA/Cho) to correctly classify those with and without ABE, was found to be excellent with an AUC of 0.992 and 0.942 for NAA/Cr and NAA/Cho respectively. As far as we are aware, no other research yielded the validity of MRS metabolic ratios for the detection of ABE.

In this research, group 1 had considerably higher absolute latencies for waves III and V than group 2 and the control group, whereas there was little variation between group 2 and healthy controls. The prolongation in III and V waves was due to an abnormality in the central auditory pathway. This is confirmed by significantly prolonged I-III and I-V interpeak intervals, that represent brainstem conduction time, in group 1 in comparison to group 2 and the control group.

Our results are quite in agreement with Ahlfors and Parker [28], Jiang et al. [29], Jiang and Wilkinson [30], and Salehi et al. [31], who observed that early prolongation of wave V latency, wave III latency, I-III and I-V interpeak intervals happened in moderate hyperbilirubinemia.

Salehi et al. [31] enrolled 42-term neonates with hyperbilirubinemia. The recording of ABR was made shortly after confirming that the total serum bilirubin level was greater than 15 μg/dl. They found a significant increase in the absolute latencies of waves III and V and interpeak latencies of I-III and I-V of the sample group compared to the normal control group (P < 0.05).

Biochemical and physiological evidence introduces synapses as the primary target for bilirubin effects. Thus, synapses along the auditory brainstem pathway can be disturbed severely [32]. This is supported by prolongation in I-III and I-V interpeak latencies, which were found in this study.

Our findings are consistent with Okhravi et al. [33], who recruited 97 terms and near-term (35–37 weeks) infants with pathologic hyperbilirubinemia (values of serum bilirubin ≥ 18 mg/dL), ABR was performed at hospital discharge, revealing that the mean latencies of waves III and V of ABR were substantially longer in the pathologic hyperbilirubinemia group than in the healthy controls (P < 0.001). Additionally, the pathologic hyperbilirubinemia group had substantially longer mean interpeak intervals for waves I-III and I-V of the ABR compared to the controls (P < 0.001).

Based on our work, we agree with Adebami [34] and Usman et al. [32] that abnormal ABR results signify the presence of ABE and are considered the most common and the earliest manifestation of ABE.

It should be noted that there is no precise TSB threshold for identifying auditory dysfunctions. The Joint Committee on Infant Hearing (JCIH), American Academy of Pediatrics (2007) had not defined any values, just referred the hyperbilirubinemia cases, in which the serum levels of total bilirubin require exchange transfusion, as risk factors for hearing loss [35].

Jiang et al. [29] suggested that TSB levels over 10 mg/dL had a detrimental impact on a newborn's auditory function and that when TSB levels got higher, waves III and V's latencies and the I-V interval tended to increase.

Esmaeilnia et al*.* [36] studied ABR changes in 51-term jaundiced neonates who had a normal newborn examination and normal otoacoustic emission (OAE) with total TSB > 15 mg/dL after the third day of birth. They demonstrated that 26.7% (4 cases from a total of 15 cases) of neonates with TSB ≥ 19 mg/dL had abnormal ABR (prolonged wave latencies and/or interpeak intervals), while 30.60% (11 cases out of 26 cases) in neonates with TSB < 19 mg/dL had abnormal ABR. Also, they found that 27.5% (11 cases from a total of 40 cases) of neonates with TSB ≥ 16 mg/dL had abnormal ABR, while among neonates with TSB < 16 mg/dL, 36.4% (4 cases out of 11 cases) showed abnormal ABR.

Like Esmaeilnia et al*.* [36] and Jiang et al. [29], we focused on the possibility of hearing damage at a lower level of hyperbilirubinemia. It appears that auditory neuropathy may occur at lower TBS concentrations that are widely used for therapeutic interventions, as according to this current study, the minimum TB level for ABR affection was 16 mg/dl (raised auditory threshold and prolonged I-III interpeak interval).

In the present study, the area under the curve (AUC) of the ROC curve for wave V peak latencies and I-V interpeak interval were 0.993 and 0.939. Thus, they were excellent detectors of bilirubin toxicity to the auditory system. To the best of our knowledge, the validity (AUC, sensitivity, specificity) of ABR parameters, including wave V peak latencies and interpeak I-V intervals for the detection of ABE, have not been demonstrated by any other researchers.

## Conclusions

Normal MRI was also seen in infants who were presented with acute bilirubin encephalopathy. Comparing the ABE group to the neonatal hyperbilirubinemia group, ^1^H-MRS indicated significantly reduced NAA/Cr and NAA/ Cho ratios with considerably elevated Lac/Cr ratios in the basal ganglia. ABR showed significantly prolonged wave V peak latency, wave III peak latency, I-III and I-V interpeak intervals among neonates of the ABE group in comparison with the neonatal hyperbilirubinemia group. MRS and ABR can be used as predictor tools for acute bilirubin encephalopathy.

## Limitations

1. Small sample size.
2. No correction for the gestational age of the studied groups.
3. ABR was recommended in the 3^rd^ month but was not followed up.

## Data Availability

The corresponding author may provide the datasets used and/or analyzed during the present investigation as MS Excel files (.xlsx) upon reasonable request.

## Abbreviations

Glu: Glutamate
MRS: Magnetic resonance spectroscopy
ANSD: Auditory neuropathy spectrum disorder
ABE: Acute bilirubin encephalopathy
ABR: Auditory brainstem response audiometry
AGA: Appropriate for gestational age
NICU: Neonatal intensive care unit
BIND-M: Bilirubin-induced neurologic dysfunction
CNS: Central nervous system
TSB: Total serum bilirubin
TEOAE: Transient evoked otoacoustic emissions
ENT: Ear, nose, and throat
MRI: Magnetic resonance imaging
FLAIR: Fluid attenuation inversion recovery
DWI: Diffusion-weighted images
CSF: Cerebro-spinal fluid
MRSI: Magnetic resonance spectroscopic imaging
PRESS: Point-resolved spectroscopy
TR: Repetition time
TE: Echo time
Cho: Choline
NAA: N-acetyl-aspartate
Cr: Creatine
Lac: Lactate
IQR: Interquartile range
SD: Standard deviation
ANOVA: Analysis of variance
ROC curve: Receiver operating characteristic curve
B/A ratio: Bilirubin/albumin ratio
PPV: Positive predictive values
NPV: Negative predictive values
HIE: Hypoxic ischemic encephalopathy
JCIH: Joint Committee on Infant Hearing
OAE: Otoacoustic emission
AUC: Area under the curve
